# *BmSV2A* and *BmSV2B* Are Involved in Regulating GABAergic Neuron-Related Gene Expression in the Silkworm, *Bombyx mori*

**DOI:** 10.3390/insects16030251

**Published:** 2025-03-01

**Authors:** Zhongyi Liu, Nur Fazleen Binti Idris, Lulu Liu, Chunping Hou, Chunyan Yang, Chengyu Zhan, Shubo Liang, Jianghong Shen, Kunpeng Lu, Hai Hu, Fangyin Dai, Xiaoling Tong

**Affiliations:** 1 State Key Laboratory of Resource Insects, Institute of Sericulture and Systems Biology, Southwest University, Chongqing 400715, China; zyliuswu@163.com (Z.L.); nurfazleenidris@gmail.com (N.F.B.I.); liululuswu@163.com (L.L.); hcp235699@163.com (C.H.); 18284172139@163.com (C.Y.); zhancyres@email.swu.edu.cn (C.Z.); shuboliang@email.swu.edu.cn (S.L.); sjh0522@email.swu.edu.cn (J.S.); lukunpeng@swu.edu.cn (K.L.); huhaiswu@163.com (H.H.); 2 Key Laboratory of Sericultural Biology and Genetic Breeding, Ministry of Agriculture and Rural Affairs, College of Sericulture, Textile and Biomass Sciences, Southwest University, Chongqing 400715, China

**Keywords:** voltinism, diapause, GABAergic neuron, synaptic vesicle glycoprotein, silkworm

## Abstract

In insects, the number of life cycles varies inter- and intra-specifically, and it is widely accepted that the variation in the number of life cycles is an adaptive response to diverse environmental conditions. However, the molecular mechanism that underlies the variety and plasticity in the number of life cycles is largely unknown. In the silkworm, *Bombyx mori*, the *Voltinism* (*V*) locus has three alleles, *V*^1^(univoltine; dominant), *V*^2^ (bivoltine; standard), and *V*^3^ (polyvoltine; recessive), which are known to generate variation in the number of life cycles in a year under natural conditions, with obligatory diapause for the *V*^1^ allele, facultative diapause for *V*^2^, and non-diapause for *V*^3^. Here, we further confirm that the γ-aminobutyric acid (GABA)ergic neuron signal pathway modulates progeny diapause via controlling diapause hormone release. A population genetic analysis (*F*st) revealed that the *synaptic vesicle glycoprotein 2A* and *2B* (*BmSV2A* and *BmSV2B*) genes, tightly related to the transport of neurotransmitters, are located in the *V* locus. Importantly, using the CRISPR/Cas9 editing technique, we have discovered that the *BmSV2A* and *BmSV2B* genes increased or modified the expression of GABAergic neuron signal pathway genes, respectively. These results demonstrate that *BmSV2A* and *BmSV2B*, positioned within the *V* locus, could be involved in voltinism control via the GABAergic neuron signal pathway.

## Simple Summary

This study reveals the genetic basis of silkworm voltinism. Based on our research findings, we have conclusively demonstrated that the GABAergic neuron signal pathway is pivotal in regulating silkworm voltinism. Thereafter, population genetic analysis was conducted on SNP variations in 109 silkworm strains using silkworm pan-genome datasets and classical genetic mapping of *V* genes (the main gene controlling voltinism). We identified four candidate genes related to silkworm voltinism. Using CRISPR/Cas9-mediated gene editing technology, we verified that the two genes, *BmSV2A* and *BmSV2B*, may participate in voltinism control by regulating gene expression in the GABAergic neuron signal pathway. Our research provides a new perspective for revealing the genetic basis of silkworm voltinism.

## 1. Introduction

**Figure 1:**
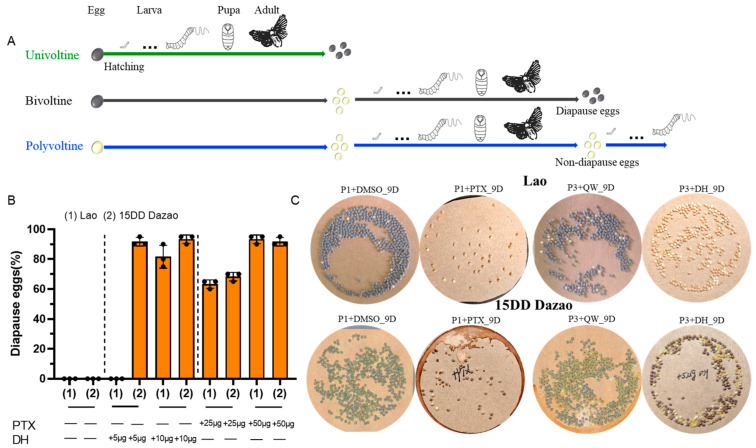
Changes in diapause rate caused by injection of diapause hormone (DH) or picrotoxin (PTX). (**A**) The number of life cycles per year in different voltine strains: univoltine (one generation), bivoltine (two generations), and polyvoltine (more than three generations). The brown eggs represent diapause eggs, which can hatch after diapause termination; the yellow eggs represent non-diapause eggs, which can hatch directly without undergoing diapause. (**B**) The effects of diapause hormone (DH) or picrotoxin (PTX) injections during the pupal stage in Lao (1) and 15DD Dazao (2) strains on offspring diapause: +25 μg or +50 μg PTX: injection of +25 μg or 50 μg PTX into 1-day-old pupae; +5 μg or +10 μg DH: injection of +5 μg or 10 μg DH into 3-day-old pupae. Diapause egg-inducing activity is represented as the percentage of oviposited eggs in each batch (*n* = 3). (**C**) Representative egg circles produced by 15DD Dazao and Lao after injection of 10 μg DH or 50 μg PTX. Every circle of eggs was laid by a single female moth. P1+DMSO_9D: injection of DMSO into 1-day-old pupae, 9 days after the eggs were laid (fully developed embryo); P1+PTX_9D: injection of PTX into 1-day-old pupae, 9 days after the eggs were laid (already diapaused); P3+QW_9D: injection of Q water into 3-day-old pupae, 9 days after the eggs were laid (fully developed embryo); P3+DH_9D: injection of DH into 3-day-old pupae, 9 days after the eggs were laid (already diapaused).

Insects are one of the most prosperous group of animals on earth, distributed in every corner of the world. To adapt to unfavorable environmental conditions and synchronize populations, most insect species evolved several important survival strategies such as migration, dormancy, and diapause [1,2,3]. Diapause is a programmed state of development suppression that insects enter prior to onset of adverse environmental conditions [4]. According to the various stages of occurrence, insect diapause can be divided into four types: embryonic, larval, pupal, and adult diapause [5,6,7]. The silkworm, *Bombyx mori*, is known as a typical embryonic diapause insect, and its strains produce one (obligatory diapause), two (facultative diapause), and more than three (non-diapause) generations per year under natural conditions, which are defined as univoltine (*V*^1^), bivoltine (*V*^2^), and polyvoltine (*V*^3^), respectively (Figure 1A) [8,9,10,11].

The key hormone regulating silkworm diapause at the molecular level is diapause hormone (DH). In the 1850s, scientists discovered that a substance secreted by suboesophageal ganglion (SG) during the pupal stage could induce offspring embryo to enter diapause, and this substance was named DH [12]. Subsequently, DH was isolated and purified from the brain (Br)–suboesophageal ganglion (SG) complex, which is a member of the FXPRLa neuropeptide family composed of 24 amino acids [13,14,15]. The diapause hormone–pheromone biosynthesis activating neuropeptide gene, *DH-PBAN,* encodes a polyprotein precursor containing DH, PBAN, α-, β-, and γ-SGNP [16,17]. Further research has shown that DH acts on a DH receptor (DHR) in the developing ovaries of females during the pupal stage and is produced in seven pairs of neurosecretory cells (DH-PBAN-producing neurosecretory cells [DHPCs]) located within the SG, transported to corpora cardiaca (CC) by nervi corporis cardiaci, and ultimately released into hemolymph [18,19]. After DH binds to the DHR in the ovaries, increasing the activity of trehalase, trehalose is converted to glucose and enters the ovaries, ultimately promoting the accumulation of glycogen in the ovaries (Figure S5A) [19,20]. Then, the glycogen is converted into glycerol and sorbitol, which are resistant to low temperatures and promote the onset of diapause [21].

GABAergic neurons determine silkworm diapause by regulating the release of DH. In the 1850s, Fukuda and Hasegawa used methods such as pupae brain removal, transplantation, or cutting of nerve cords to confirm that the brain can control the release of DH from the SG [22]. Since the discovery of γ-aminobutyric acid (GABA) in the brain, it has been considered a major inhibitory neurotransmitter [23,24,25]. Initially, research reported that GABA was injected into pupae that produced diapause eggs after metamorphosis, successfully inducing female moths to lay some non-diapause eggs. By injecting the GABA receptor antagonist picrotoxin (PTX) into pupae producing non-diapause eggs, researchers modified voltinism by producing partial diapause eggs [26,27,28]. Subsequently, some studies also showed that GABAergic neurons control silkworm diapause by regulating the amount of DH release [29,30]. The currently available evidence clearly indicates that the diapause of silkworms is closely related to the GABAergic neuron signal pathway.

To date, the genetic basis of silkworm voltinism remains poorly understood. Previous classical genetic studies mapped three multiallelic loci (*V*^1^, *V*^2^, *V*^3^) at 42.6 cM on chromosome 6, which govern uni-, bi-, and polyvoltine phenotypes, exhibiting a hierarchical dominant–recessive allelic relationship (*V*^1^ > *V*^2^ > *V*^3^). Using an integrative genetic approach that combines population genetic analysis with classical linkage mapping of *V* locus, we localized the voltinism-related genes *BmSV2A* and *BmSV2B* in the silkworm, *Bombyx mori*. Importantly, we demonstrated that *BmSV2A* and *BmSV2B* could control silkworm voltinism by regulating the expression levels of GABAergic neuron-related genes.

## 2. Materials and Methods

### 2.1. Silkworm Strains

All experimental strains were provided by the National Silkworm Genetic Resources Bank (Southwest University, Chongqing, China). Lao (*V*^3^) was used and incubated under full light at 25 °C (non-diapause), while Dazao (*V*^2^) was incubated under full light at 25 °C (25LL, diapause) and full darkness at 15 °C (15DD, non-diapause), respectively. The larvae of all strains were raised in an environment with a room temperature of 25 °C (±1.5 °C) and relative humidity of 75–85%, under natural light, and fed fresh mulberry leaves at regular intervals every day. Pupae used in this experiment were collected within 2 h after ecdysis (referred to as P0) to synchronize their subsequent development, and pupae were kept at 25 °C until eclosion.

### 2.2. Injection of PTX and DH

Totals of 25 μg or 50 μg PTX (CAS No.: 124–87-8, MedChemExpress, Monmouth Junction, NJ, USA) and DMSO (control group) were injected into the first stomata of 1-day-old pupae (24 ± 2h). A total of 5 μg or 10 μg DH (CAS No.: P28494, Sangon Biotech, Shanghai, China) was injected into the fourth stomata of 3-day-old pupae (72 ± 2h), with Milli-Q water used as the control group. The areas around the stomata and epidermis of the pupae abdomen were wiped with 75% alcohol before and after injections as a sterilization technique. The injected pupae were kept at 25 °C, development was observed, and the rate of offspring diapause was recorded.

### 2.3. Ehrlich’s Diazo Reaction

As previously reported, Ehrlich’s diazo reagent was used to detect 3-hydroxykynurenine [30]. The reagent preparation and experimental procedure are as follows. Solution A: place 0.45 g of sulfanilic acid (CSA No.: 121–57-3, MACKLIN, Shanghai, China) into a 50 mL centrifuge tube, add a small amount of Q water to dissolve it, and then add 4.5 mL of concentrated hydrochloric acid. Finally, adjust the volume to 50 mL with Q water. Solution B (5% NaNO_2_ solution): 2.5 g of NaNO_2_ was dissolved in 50 mL of Q water. Solution C (3% Na_2_CO_3_ solution): 1.5 g of Na_2_CO_3_ was dissolved in 50 mL of Q water.

AB mixture: 750 μL of solution A and 750 μL of solution B were mixed and placed on ice for 5 min. A total of 3 mL of solution B was added into the above mixture and placed for 5 min, and finally 20.5 mL of precooled Q water was added. The final mixture was placed on ice for 5 min (to keep it cold until usage).

On the day of adult emergence, complete ovaries (including eggs) were dissected from the moth and washed with PBS. The samples were dried on filter paper, and 100 mg of ovaries were homogenized with 3% Na_2_CO_3_ solution (500 μL). After centrifugation (9600× *g* for 5 min), the supernatant was collected and the sample solution was tested. A total of 250 μL of supernatant was taken, and then 250 μL of the Na_2_CO_3_ solution and 200 μL of the AB mixture were added. The mixture was mixed well and the color was observed and recorded.

### 2.4. Immunofluorescence of the Pupal Brain–Suboesophageal Ganglion Complex

Immunoreaction procedures were adapted from the protocol for immunofluorescent staining of the *Drosophila* larval brain [31]. Briefly, the brain–suboesophageal ganglion (Br-SG) complex was dissected and fixed in fixative solution containing 4% paraformaldehyde and incubated at 4 °C overnight. The fixed tissues were washed with PBS containing 0.2% Tween-20 (PBSTw). Tissue samples were soaked in PBS containing 2% Triton X-100 (PBSTr) at 25 °C for 6 h; the tissues were washed with PBSTw, blocked with PBSTw containing 5% heat-inactivated goat serum and 2% BSA, and incubated with anti-DH[N] (ABclonal Technology, Wuhan, China) at 1:500 at 4 °C overnight. The signal was detected with Cy3-labeled IgG (CAS No.: A0521, Beyotime Biotechnology, Shanghai, China) diluted to 1:500 and the images were viewed using the Research Biological Microscope (BX63, Olympus Corporation, Shinjuku, Japan).

### 2.5. RNA Extraction and qRT-PCR Analysis

The total RNA of each sample was extracted using the MicroElute Total RNA Kit (R6831, Omega, Mountain Lakes, NJ, USA) according to the manufacturer’s protocol. The PrimeScript RT reagent kit with gDNA Eraser (RR047, Takara, Kusatsu, Japan) was used for the reverse transcription. Quantitative RT-PCR (qRT-PCR) analyses were performed using the qTOWER^3^ Real Time PCR System (Analytik jena, Jena, Germany) with the SYBR Green Premix *Pro Taq*HS qPCR Kit (Accurate Biology, Guangzhou, China) according to the manufacturer’s instructions. The relative mRNA levels of the target genes were calculated using the 2^−∆∆Ct^ method and the qRT-PCR protocol was as follows: denaturation at 95 °C for 30 s followed by 40 cycles of 95 °C for 5 s and 60 °C for 30 s. The silkworm *ribosomal protein 49* (*Rp49*) gene was used as the internal control. The primers used for qRT-PCR are listed in Table S1.

### 2.6. CRISPR/Cas9-Mediated Gene Knockout

Standard protocols were used for gene knockout, as previously described [32]. The genes’ particular sgRNA target sites were screened using the online tool CRISPRdirect (http://crispr.dbcls.jp/, accessed on 28 September 2022). Two sgRNA target sites were designed for each gene to ensure gene editing efficiency. The sgRNA target sequences (20 mer + PAM) and PCR detection primers for each knockout line are listed in Table S2. The knockout lines were obtained by injecting the mixture of Cas9 protein and sgRNA, and homozygous mutants were screened. SgRNAs were synthesized using the TranscriptAid T7 High Yield Transcription Kit (K0441, Thermo, USA) and then diluted to 1000 ng/μL. Nine microliters of sgRNA were added to 1 μL of TrueCut Cas9 Protein v2 (A36499, Invitrogen, Waltham, MA, USA) and mixed gently. The mixture was incubated at 37 °C for 15 min and then used for microinjection, where the amount of sgRNA injected into each egg was 8–10 ng. The hatched caterpillars and the offspring were raised, and the adult moths were subjected to molecular detection to determine the stable genetic lines using PCR and DNA sequencing.

### 2.7. Annotation of Genes in V Locus

All gene annotation and physical location information were retrieved from the Silkworm Pan-genome Database [33] (SilkMeta, http://silkmeta.org.cn, accessed on 15 September 2022). Domain analysis and functional annotation of proteins were carried out with the online tools of SMART (https://smart.embl.de/, accessed on 23 September 2022) and NCBI blast (https://blast.ncbi.nlm.nih.gov, accessed on 23 September 2022).

### 2.8. Population Divergence Index (Fst)

Through the “Silkworm Pan-genome Project”, our team has successfully completed the deep resequencing of 1078 silkworm strains. With the help of the National Silkworm Genetic Resource Bank (Chongqing, China), we investigated the voltinism of 1078 silkworm strains and ultimately selected 109 strains with confirmed voltinism for population genetic analysis. The 109 strains included 35 univoltine strains, 56 bivoltine strains, and 18 polyvoltine strains (Table S3). Genomic data for these strains were downloaded from SilkMeta. Voltinism-related regions were estimated using a sliding window approach, with a 5 kb window and 5 kb step size. For each window, we calculated the population divergence index (*F*st) based on the prior report [33].

## 3. Results

### 3.1. GABAergic Neurons Control Silkworm Diapause by Regulating the Release of DH in the Subesophageal Ganglion

Given that GABAergic neurons play a key role in the regulation of diapause, we further analyzed the effect of GABAergic neurons on diapause in the *V*^2^ (Dazao incubated at 15 °C, non-diapause) and *V*^3^ (Lao, non-diapause) strains. To achieve this goal, DMSO (employed as a control since PTX is insoluble in Q water yet soluble in DMSO) and PTX, which functioned as an inhibitor of GABAergic neurons, were injected into 1-day-old pupae of the non-diapause egg producer. Consistent with previously reported results, we found that the moths of the non-diapause egg producer Lao and 15DD Dazao were able to lay diapause eggs after injection of PTX (Figure 1B,C). However, the number of eggs laid significantly decreased (Figure S1). At the same time, we discovered that when DH was injected into the 3-day-old pupae of the non-diapause egg producers, specifically Lao and 15DD Dazao, it also induced the moths to lay diapause eggs. Here, the injected Q water group served as the control, as the purchased DH dissolves in Q water. Based on the above results, we speculated that DH, synthesized in the SG of the pupae of Lao and 15DD Dazao, was released into hemolymph. This release occurred because the PTX inhibited the signal pathway of GABAergic neurons, ultimately leading the moths to lay diapause eggs.

**Figure 2:**
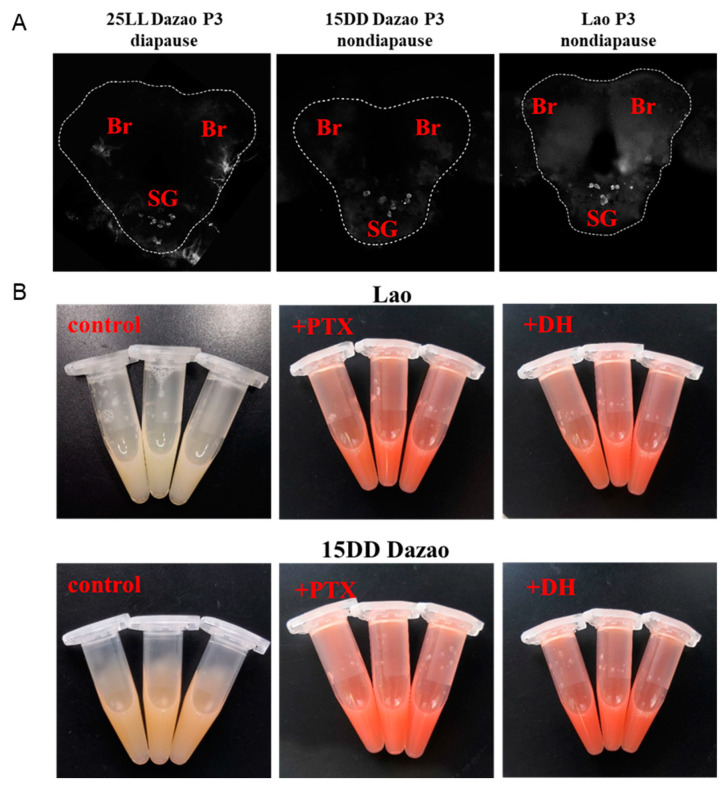
Inhibition of GABAergic neuron signals leads to DH release from the SG into hemolymph during the pupal stage. (**A**) Diapause hormone (DH) content in brain–suboesophageal ganglion (Br-SG) immunofluorescence views: 25LL Dazao P3: immunofluorescence of the Br-SG of Dazao pupae (the Dazao strain was incubated under full light conditions at 25 °C, diapause); Lao P3: immunofluorescence of Br-SG of the Lao polyvoltine strain (the Lao strain was incubated under full light conditions at 25 °C, non-diapause); 15DD Dazao P3: immunofluorescence of the Br-SG of Dazao (the Dazao strain was incubated under full dark conditions at 15 °C, non-diapause). All Br-SG were taken at the 3-day-old pupal stage. Br: pupal brain; SG: pupal suboesophageal ganglion. White-dashed box: an outline of the brain–suboesophageal ganglion. (**B**) Effect of PTX or DH injection in Lao and 15DD Dazao strains on 3-hydroxykynurenine accumulation levels in the ovaries. Lao control: representative color reaction of the solution after injection of DMSO (control group) into 1-day-old pupae; +PTX: representative color reaction of the solution after injection of PTX into 1-day-old pupae; 15DD Dazao control: representative color reaction of the solution after injection with Q water (control group) into 3-day-old pupae; +DH: representative color reaction of the solution after injection of DH into 3-day-old pupae.

To verify this hypothesis, the Br-SG complex of the 3-day-old pupae of Lao and 15DD Dazao was dissected and immunofluorescence staining was performed to examine the presence of DH in the Br-SG complex. A large amount of DH in the SG was observed under an upright fluorescence Research Biological Microscope (BX63, Olympus Corporation, Japan) (Figure 2A). DH causes the accumulation of 3-hydroxykynurenine in the ovaries, which is a precursor to the ommochrome pigments in the serosal membrane of diapause eggs. [26,34]. There have been studies reporting that the reaction between Ehrlich’s diazo reagent and 3-hydroxykynurenine produces a red color [28,30]. To indirectly evaluate DH content in hemolymph, we carefully dissected the ovaries on the day of adult emergence and the color of reaction solutions was recorded, representing the amount of 3-hydroxykynurenine in the ovaries. The results were consistent with our expectations. Ehrlich’s diazo reaction solutions were light yellow in the control groups (normal Lao and 15DD Dazao, non-diapause), whereas reaction solutions turned dark red after injection of PTX or DH (Figure 2B). These results indicated that DH was released from the SG into hemolymph after injection of PTX into Lao and 15DD Dazao. In summary, we found that the non-diapause egg producer (Lao and 15DD Dazao) synthesized relatively the same amount of DH as the diapause egg producer (25LL Dazao), but the DH was not being released into hemolymph during the pupal stage. This result showed that GABAergic neuron could be one of the main factors controlling the amount of DH release from the SG into hemolymph.

### 3.2. Identification of Loci Related to Voltinism Using the Pan-Genome

**Figure 3:**
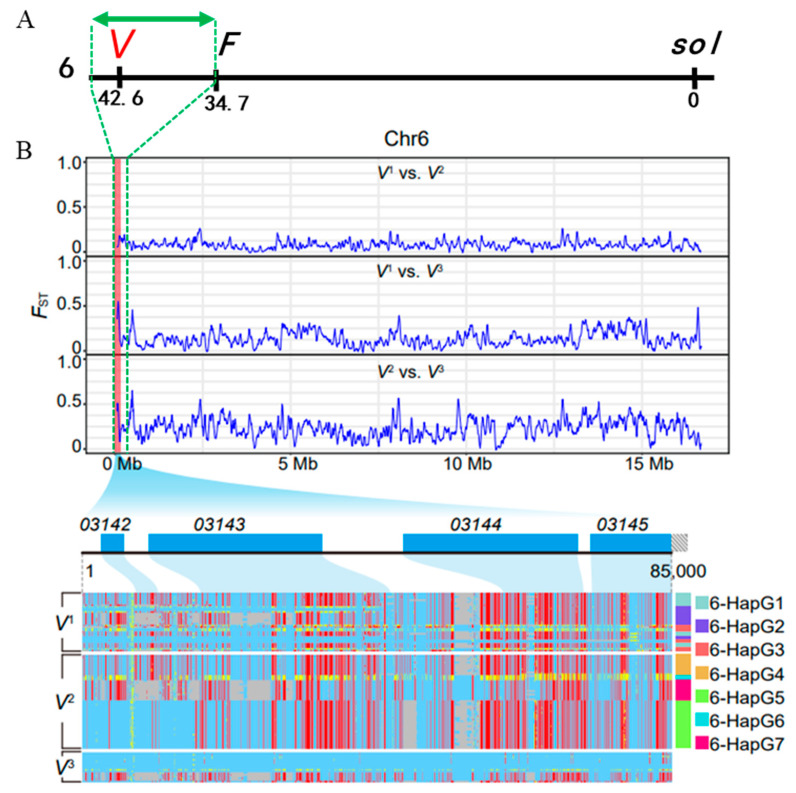
Localization of voltinism-related genes. (**A**) Classical genetic mapping of the main gene *V* controlling voltinism in silkworm on chromosome 6 (loci 42.6). (**B**) Population genetic differentiation index *F*st and candidate genome region heatmap among different voltine groups. *V*^1^: univoltine group; *V*^2^: bivoltine group; *V*^3^: polyvoltine group; 6-HapGX: the haplotype GX of chromosome 6 in this region (X = 1, 2, …, 7).

In the silkworm, there are strains that produce one, two, or more than three generations per year under natural conditions, and these numbers are determined by allelic variation at a single chomosome 6 locus, known as *Voltinism* (*V*). The population genetic analysis using silkworm pan-genome data, including samples of *V*^1^, *V*^2^, and *V*^3^, was performed in order to identify voltinism-related genes at the *V* locus (Table S3) [33]. We found that the genomic region between the previously reported *Flesh* (*F*) locus (6–34.7 cM, *KWMTBOMO03155*) [35] and the end of chromosome 6 shows a strong positive signal (Figure 3A,B). This region within the *V* locus includes the genes *KWMTBOMO03142*, *KWMTBOMO03143*, *KWMTBOMO03144*, and *KWMTBOMO03145*. According to the gene function annotation referring to the Silkworm Pan-genome Database website (Table S4), we surprisingly found that the genes *KWMTBOMO03143* and *KWMTBOMO03144* were predicted to be functioning for the encoding of synaptic vesicle glycoproteins 2B (BmSV2B) and 2A (BmSV2A), which belong to the major facilitator superfamily (MFS) that promotes the transmembrane transport of substances. (Figures S2B and S3B). By searching in the National Center for Biotechnology Information (NCBI) database using the Basic Local Alignment Search Tool for protein sequences (BLASTp), we found a 32.2% similarity between BmSV2A and SV2A proteins and a 35.4% similarity between BmSV2B and SV2B proteins, which showed the same MFS structural domain (Figures S2 and S3). This suggests that BmSV2A and SV2, as well as BmSV2B and SV2B, were predicted to perform similar functions in the organism. Previous studies have demonstrated that *SV2A* and *SV2B*, which are specifically expressed in synaptic vesicles, can regulate action potential-dependent neurotransmitter GABA release and the density of GABAergic neuron signals in mammals [36,37,38]. Therefore, *BmSV2A* and *BmSV2B* genes were selected as the candidate genes for this study and the functions of the genes were examined.

### 3.3. Knockout of BmSV2A and BmSV2B Affects the Expression of GABAergic Neuron Signal Pathway Genes

**Figure 4:**
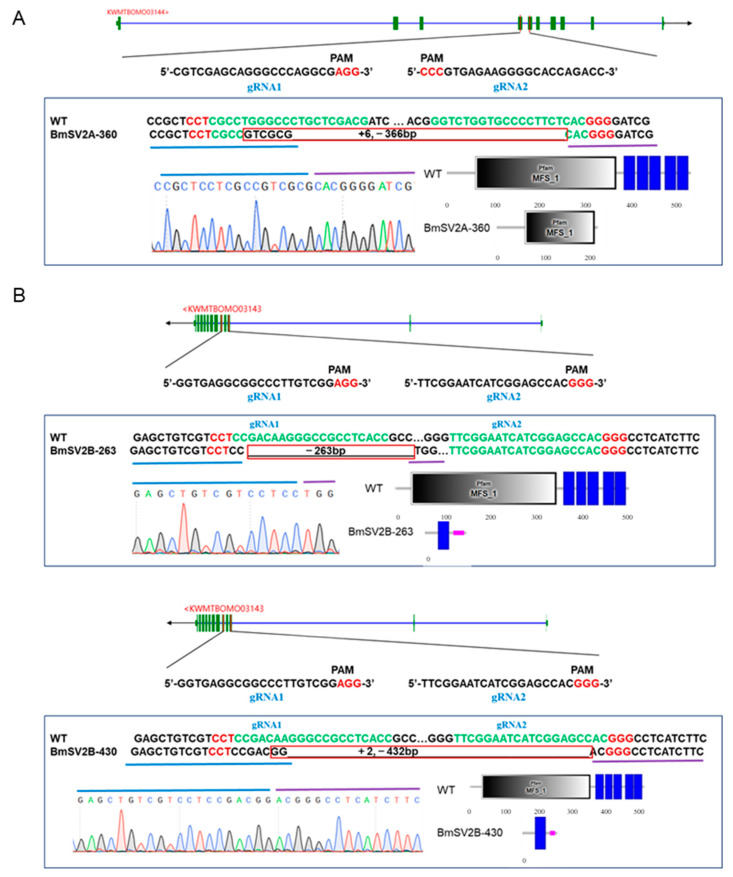
Genotypic validation of CRISPR/Cas9-mediated *BmSV2A* and *BmSV2B* knockout lines in the Lao strain. (**A**) Schematic representation of *BmSV2A* knockout in the Lao strain, depicting sgRNA target sites (top), genomic sequence variation (middle), PCR product sequencing validation (bottom left), and protein domain prediction (bottom right). The green boxes denote exons and blue lines denote introns. BmSV2A-360 represents a 360 bp deletion in the genome between the two sgRNA targeting sites (+6: insertion of 6 bp; −366: deletion of 366 bp). (**B**) Schematic representation of *BmSV2B* knockout in the Lao strain. BmSV2B-263 represents a 263 bp deletion in the genome between the two sgRNA targeting sites; BmSV2B-430 represents a 430 bp deletion in the genome between the two sgRNA targeting sites (+2: insertion of 2 bp; −432: deletion of 432 bp).

The spatio expression levels of *BmSV2A* and *BmSV2B* genes in different tissues of the polyvoltine strain Lao (*V*^3^) on 1-day-old pupae stage were evaluated. Quantitative results indicate that *BmSV2A* is highly expressed in the ovaries, body fat, and the Br-SG, and *BmSV2B* is highly expressed in the Br-SG (Figure S4). Therefore, we speculate that *BmSV2A* and *BmSV2B* may regulate GABAergic neuron signals within the central nervous system of the brain. To test the function of *BmSV2A* and *BmSV2B*, CRISPR/Cas9-mediated knockout was performed for Lao (Figure 4).

**Figure 5:**
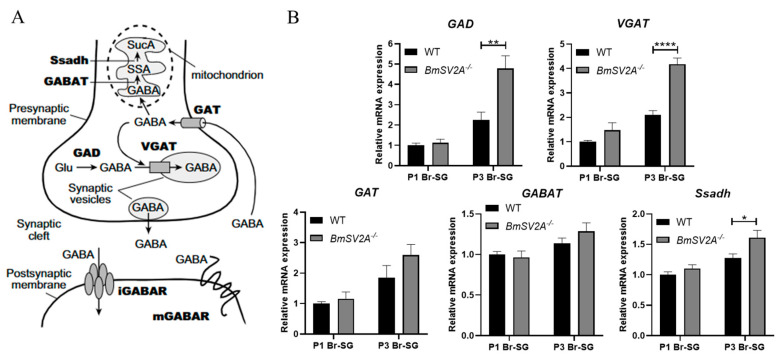
Expression of GABA synthesis, transport, and degradation enzyme genes. (**A**) Schematic overview of a GABAergic synapse, cited from Ilg et al. [39]. SucA, succinic acid; SSA, succinic semialdehyde; Ssadh, succinic semialdehyde dehydrogenase; GABAT, GABA transaminase; GAT, plasma membrane GABA transporter; VGAT, vesicular GABA transporter; GAD, glutamate decarboxylase; iGABAR, ionotropic GABAR; mGABAR, metabotropic GABAR. (**B**) Effect of *BmSV2A* knockout on *GAD*, *VGAT*, *GAT*, *GABAT*, and *Ssadh* expression in brain–SG complexes during pupal–adult development. P1 means 24 h (±2 h) after pupation and P3 means 72 h (±2 h) after pupation. Brain–SG complexes were dissected out. Each bar represents the mean ± SE of six samples. * *p* < 0.05; ** *p* < 0.01; **** *p* < 0.0001.

**Figure 6:**
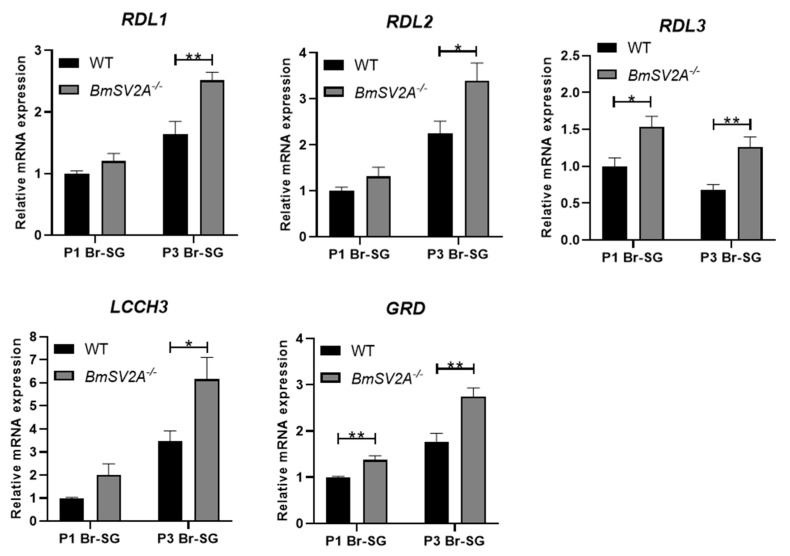
Relative GABA receptor gene mRNA levels for *RDL1*, *RDL2*, *RDL3*, *LCCH3*, and *GRD* in brain–SG complexes between Lao in *wt* and *BmSV2A* knockout mutant during pupal–adult development. Brain–SG complexes were collected 1 and 3 days after pupation (P1 and P3). Each bar represents the mean ± SE of six samples. * *p* < 0.05; ** *p* < 0.01.

*BmSV2A*^−/−^ and *BmSV2B*^−/−^ homozygote mutants were obtained and the gene expression level changes related to diapause determination pathways were evaluated. Firstly, we detected the expression levels of genes associated with DH synthesis and action signaling pathways (Figure S5A). Interestingly, in the *BmSV2A* knockout strains, the transcriptional regulatory factor *Pitx* of *DH-PBAN* showed significantly upregulated expression in Br-SG at P1 and P2 stage (Figure S5B). However, there was no significant difference in the expression of *DH-PBAN*, *DHR* and *Treh-2* genes in the ovaries (Figure S5B). Secondly, the expression of GABAergic neuron signal pathway related genes that control the amount of DH release was investigated (Figure 5A). Surprisingly, in the *BmSV2A* knockout strains, the expression levels of GABA synthesis and transport pathway genes *GAD* and *VGAT* significantly increased (Figure 5B). Similarly, the expression levels of the five subunits of the ionic GABA receptor genes, *RDL1*, *RDL2*, *RDL3*, *LCCH3*, and *GAD*, were significantly upregulated (Figure 6).

**Figure 7:**
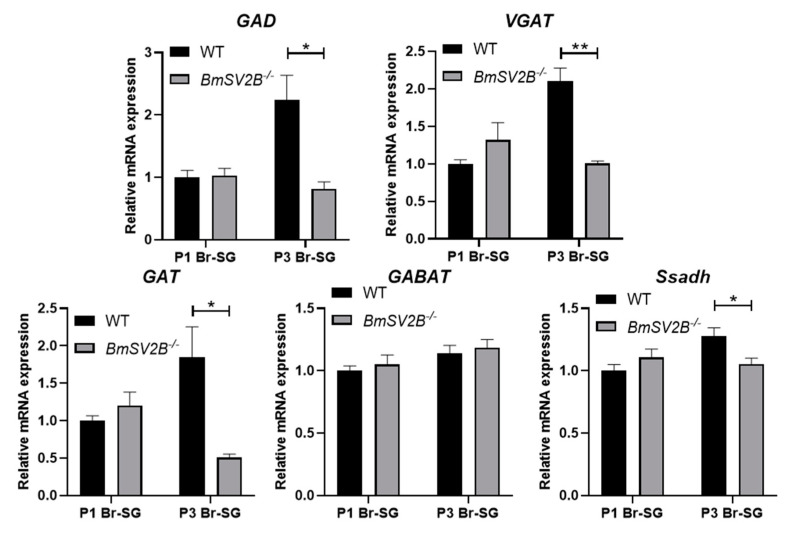
Effect of *BmSV2B* knockout on *GAD*, *VGAT*, *GAT*, *GABAT*, and *Ssadh* expression in brain–SG complexes during pupal–adult development. P1 means 24 h (±2 h) after pupation and P3 means 72 h (±2 h) after pupation. Brain–SG complexes were dissected out. Each bar represents the mean ± SE of six samples. * *p* < 0.05; ** *p* < 0.01.

**Figure 8:**
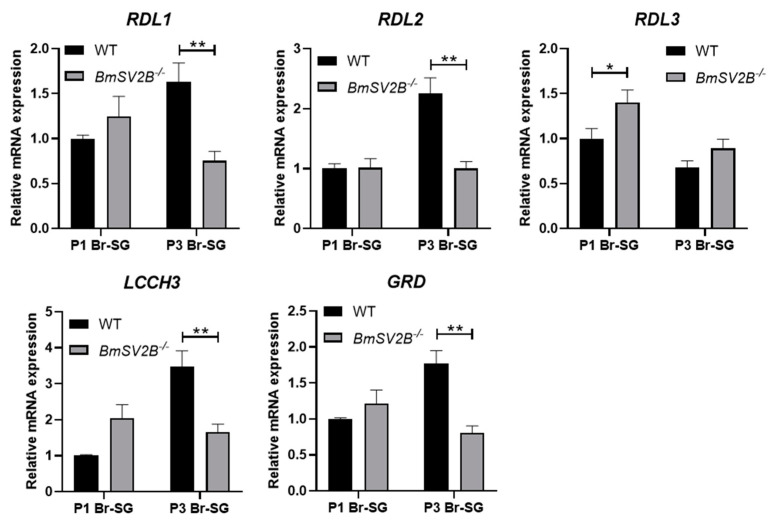
Relative GABA receptor gene mRNA levels for *RDL1*, *RDL2*, *RDL3*, *LCCH3*, and *GRD* in brain–SG complexes between Lao in *wt* and *BmSV2B* knockout mutant during pupal–adult development. The brain–SG complexes were collected 1 and 3 days after pupation (P1 and P3). Each bar represents the mean ± SE of six samples. * *p* < 0.05; ** *p* < 0.01.

At the same time, in the *BmSV2B* knockout strains, we also investigated the expression levels of the above-mentioned genes and found that the expression of the DH synthesis gene *DH-PBAN* decreased, while there was no difference in the expression levels of *Pitx*, *DHR*, and *Treh-2* genes (Figure S6). In GABAergic neuron-related signal pathways, the expression levels of GABA synthesis-related genes (*GAD* and *VGAT*) and the GABA transporter gene (*GAT*) were significantly downregulated (Figure 7). The expressions of the five subunits of the ionic GABA receptor exhibited inconsistent regulatory patterns. Among them, the expression of the *RDL3* subunit was upregulated, whereas the expressions of the *RDL1*, *RDL2*, *LCCH3*, and *GRD* subunits were downregulated (Figure 8).

In summary, *BmSV2A* knockout in the Lao strain significantly upregulated the expression of *GAD* (a GABA synthase) and *VGAT* (a vesicular GABA transporter). This elevated expression may enhance GABA synthesis and vesicular packaging, ultimately promoting more GABA release into synaptic clefts. Simultaneously, the expression levels of the five subunits of the ionotropic GABA receptor also increased significantly. These two changes could enhance the transmission of GABAergic neuron signals.

*BmSV2B* knockout in Lao strains significantly downregulated *GAD* and *VGAT* expression. This downregulated expression implies diminished GABA synthesis and vesicular packaging, ultimately reducing GABA release into synaptic clefts. Concurrently, most ionic GABA receptor subunits are downregulated, which may weaken GABAergic neuron signals. The above two changes could reduce the transmission of GABAergic neuron signals. However, this is perhaps due to the compensatory effect of the signal pathway. Reduced expression of the GABA transporter gene *GAT* likely prolonged GABA retention in the synaptic cleft, potentially amplifying its synaptic signal. Overall, knocking out *BmSV2B* disrupts the expression of GABAergic neuron signal pathway related genes, but further studies should be carried out to evaluate the genes’ function with respect to governing GABAergic neuron signal transmission.

## 4. Discussion

Silkworm voltinism varieties with different generations in a year are used as excellent models for decoding the genetic basis for diapause [29,40,41]. Previous reports presumed, through some surgical manipulation experiments during the pupal stage, that the brain could inhibit and stimulate the release of diapause hormone (DH) from the SG [42,43,44]. Furthermore, the function of GABAergic neuron with respect to controlling the release of DH was discovered in some pharmacological studies [26,27,28]. Consistent with previous findings, our data provide further proof that injection of PTX, a GABA antagonist, into non-diapause producer pupae induces DH release into hemolymph from the SG and causes the moths to lay diapause eggs (Figure 1 and Figure 2), which supports the hypothesis that DH secretion is controlled by GABAergic neuron.

Recently, research has revealed that a hierarchical pathway consisting of GABAergic and downstream corazonin (Crz) signals control DH secretion [29]. More significantly, they observed that knockout of *GAT* caused diapause egg producers to lay non-diapause eggs. At the same time, Cui et al. observed that expression levels of *GAT* and *GABAT* in the pupal Br-SG were downregulated in the circadian gene *Per* KO mutants [30]. On the other hand, the authors also found that expression levels of *GAD* and *GRD* were upregulated in the mutants. These changes in the expression level of genes caused an elevated amount of GABA in the Br-SG of pupae, which induced *Per* KO mutants to lay a significant proportion of non-diapause eggs even under diapause induction conditions. These observations suggest that the transcription levels of GABAergic neuron-related genes plays a core role in diapause determination.

An exciting finding of our research is that *synaptic vesicle glycoproteins 2A* and *2B* genes closely related to neurotransmitter secretion and synaptic plasticity are located within the *V* locus (Figure 3). These genes are assumed to be the *V* genes that govern silkworm voltinism determination. Both *SV2A* and *SV2B* are located on the synaptic vesicles and are thought to play a role in regulating the fusion of these vesicles with the presynaptic membrane, which is necessary for the release of GABA into the synaptic cleft [37]. In addition, it has been reported that synaptic vesicle glycoprotein 2A (SV2A) is a transmembrane protein of synaptic vesicles, present in all synaptic terminals, irrespective of neurotransmitter content. It is involved in key functions of neurons and focused on the regulation of neurotransmitter release [45,46,47]. Moreover, the expression of *SV2A* is ubiquitous, with stronger associations between *SV2A* andγ-aminobutyric acid (GABA)ergic synapses than glutamatergic synapses observed in some brain structures [38]. Unlike *SV2A*, *SV2B* is present in most but not all glutamatergic neurons and absent from GABAergic neurons [37]. Therefore, the functions of *SV2A* and *SV2B* are inconsistent in mammals. Similarly, the functions of *BmSV2A* and *BmSV2B* genes may also be inconsistent in the silkworm. It is worth exploring the functions of these genes in the silkworm related to the diapause mechanism via the GABAergic neuron signal pathway.

Then, we constructed homozygous mutants with knockout of the *BmSV2A* and *BmSV2B* genes, respectively (Figure 4). Our data demonstrated that expression of the GABA synthesis gene *GAD* and transport gene *VGAT* significantly increased in the *BmSV2A* KO mutant (Figure 5). In addition, the expression levels of the five subunits of the ionic GABA receptor, *RDL1*, *RDL2*, *RDL3*, *LCCH3*, and *GAD*, were significantly upregulated (Figure 6). These changes in the transcription levels of genes could lead to an increased concentration of the GABA neurotransmitter in the Br-SG [30]. Previous studies have shown that GABA, an inhibitory neurotransmitter, induced non-diapause eggs to be laid when injected into a young diapause egg producer pupal [26]. Therefore, we speculate that knocking out *BmSV2A* in a univoltine stain that only lays diapause eggs can enhance GABAergic neuron signals, leading to the laying of non-diapause eggs. Due to the limitations of current gene editing techniques, it is very difficult to obtain a *BmSV2A* KO mutant in a univoltine strain. In the future, further investigation is needed to explore the functions of *BmSV2A* to determine silkworm diapause and voltinism via the GABAergic neuron signal pathway.

In the *BmSV2B* KO mutant, our data indicated that the expression levels of genes *GAD* and *VGAT* are downregulated. This downregulated expression implies diminished GABA synthesis and vesicular packaging, thereby suppressing synaptic GABA release into the cleft. At the same time, most ionic GABA receptor subunits are downregulated, which may weaken the GABAergic neuron signal pathway. The above two changes could reduce the transmission of GABAergic neuron signals. This study demonstrates that injecting PTX into 1-day-old pupae can block the GABAergic neuron signal pathway and induce non-diapause egg producers to lay diapause eggs (Figure 1B,C). However, in the *BmSV2A* KO mutant, the above phenomenon was not observed, perhaps due to the compensatory effect of the GABAergic neuron signal pathway. The expression level of the plasma membrane GABA transporter *GAT* was significantly downregulated (Figure 7), which may prolong the residence time of GABA in the synaptic cleft, allowing previously weakened GABAergic neuron signals to still function normally. Overall, our findings suggest that *BmSV2A* and *BmSV2B* may be used as new RNAi targets for artificially controlling silkworm voltinism according to production needs.

## 5. Conclusions

In this study, we analyzed the effects of the GABAergic neuron signal pathway on progeny diapause in the *V*^2^ and *V*^3^ strains and found that blocking the GABAergic neuron signal pathway in non-diapause egg producers at the pupal stage induced them to lay diapause eggs. Subsequently, based on the silkworm pan-genome, we mapped genes *BmSV2A* and *BmSV2B* within the *V* locus on chromosome 6 through population genetic analysis (*F*st). Then, we verified the function of genes *BmSV2A* and *BmSVB* in the *V*^3^ strains (Lao) using CRISPR/Cas9-mediated gene editing technology and found that knockout of *BmSV2A* and *BmSV2B* could enhance or disturb GABAergic neuron signals, respectively. This is the first report regarding the function of *BmSV2A* and *BmSV2B* in the GABAergic neuron signal pathway, providing a new perspective on the decoding of the genetic basis of silkworm voltinism.

## Data Availability

Most of the analytical data are provided in the article. More original datasets used and analyzed during the current study are available from the corresponding author upon reasonable request.

## Supplementary Materials

The following supporting information can be downloaded at: https://www.mdpi.com/article/10.3390/insects16030251/s1, Figure S1: The number of eggs were oviposited from Lao and 15DD Dazao; Figure S2: Sequence alignment and functional domain prediction of BmSV2A and SV2A proteins; Figure S3: Sequence alignment and functional domain prediction of BmSV2B and SV2B proteins. Figure S4: Tissue expression profiles of *BmSV2A* and *BmSV2B* genes. Figure S5: Expression of genes related to the synthesis and action pathways of diapause hormone. Figure S6: Expression of genes related to the synthesis and action pathways of diapause hormone. Table S1: Primers for qRT-PCR. Table S2: SgRNA target sequences and PCR detection primers for each knockout line. Table S3: Analysis materials for population genetic differentiation index *F*st. Table S4: Candidate region encoding protein genes and functional annotations.
