# Supplementary material for: BmSV2A and BmSV2B Are Involved in Regulating GABAergic Neuron-Related Gene Expression in the Silkworm, Bombyx mori
Source: Insects. 2025 Mar 1;16(3):251. doi: 10.3390/insects16030251 (PMC11943286; doi:10.3390/insects16030251)
Supplement: Supplementary file 1 [file insects-16-00251-s001.zip › insects-3453426-supplementary.pdf]

insects

MDPI

Supplementary Materials

Figures S1–S6

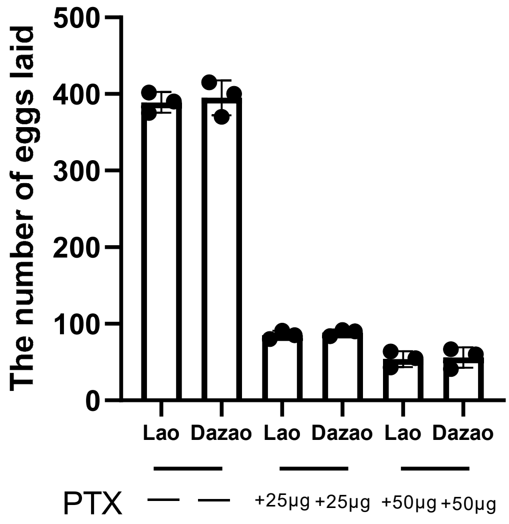

Figure S1. The number of eggs were oviposited from Lao and 15DD Dazao. +25 μg means injected 25 μg PTX into 1-day-old pupae. +50 μg means injected 50 μg PTX into 1-day-old pupae. Thereafter, count the number of eggs laid by moths after injecting PTX during the pupal stage.

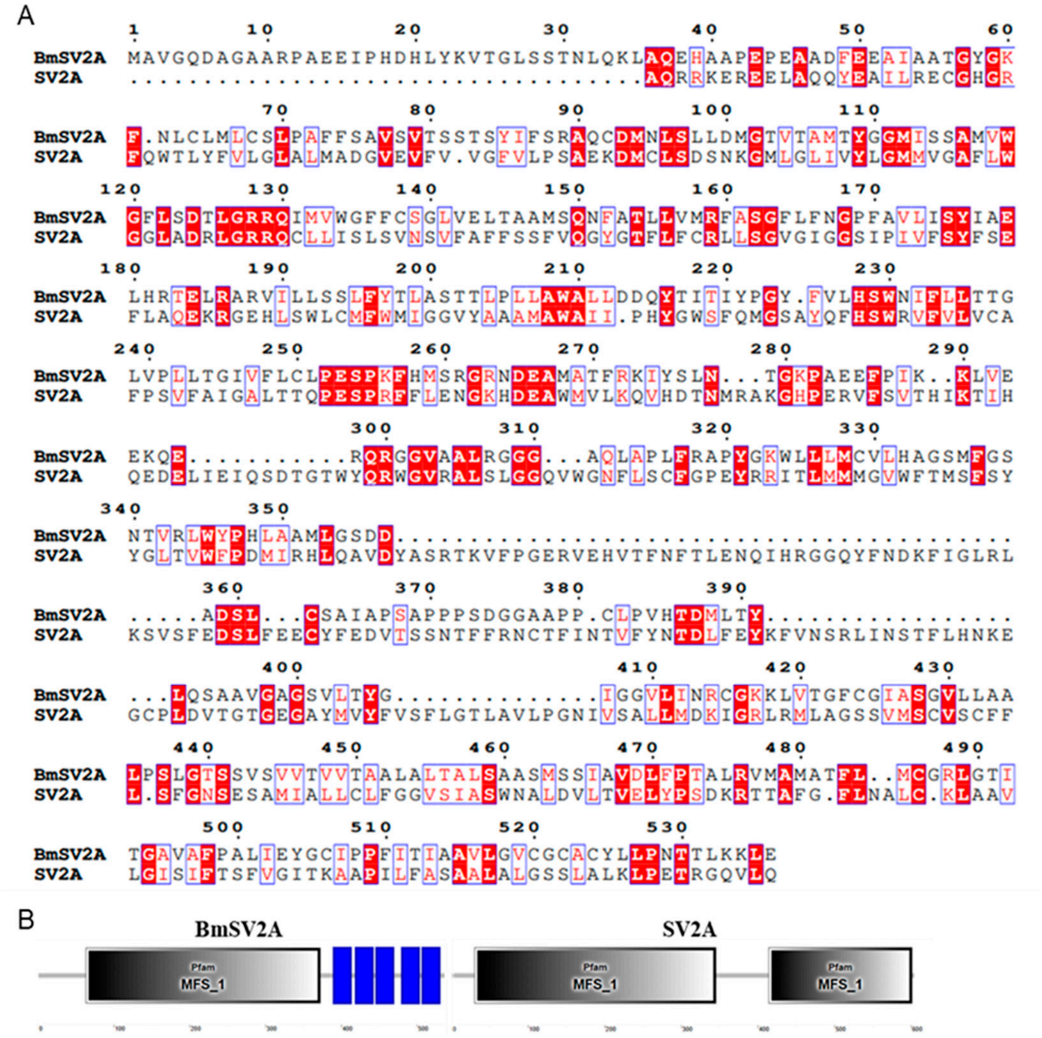

Figure S2. Sequence alignment and functional domain prediction of BmSV2A and SV2A proteins. (**A**) BmSV2A and SV2A protein sequence alignment. (**B**) Functional domain of BmSV2A and SV2A protein.

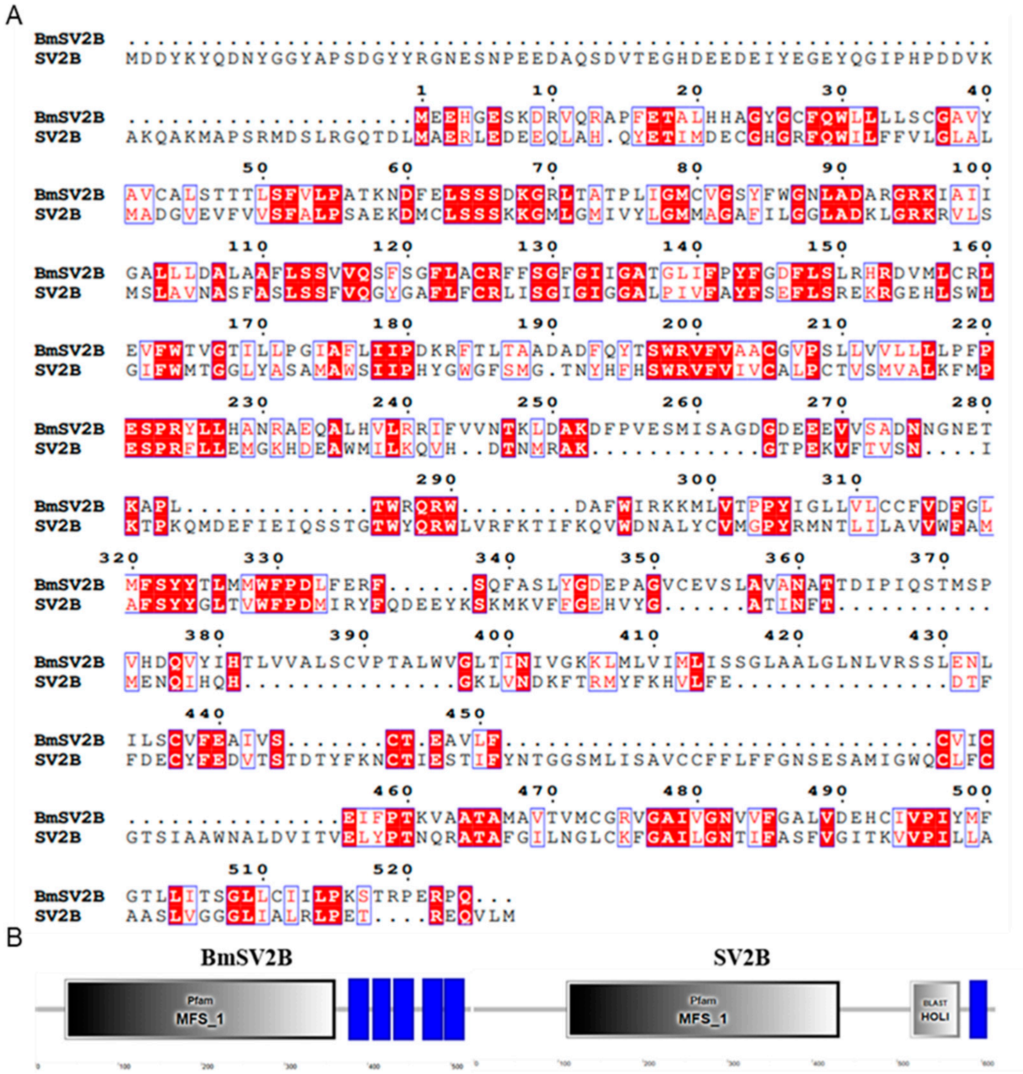

Figure S3. Sequence alignment and functional domain prediction of BmSV2B and SV2B proteins. (**A**) BmSV2B and SV2B protein sequence alignment. (**B**) Functional domain of BmSV2B and SV2B protein.

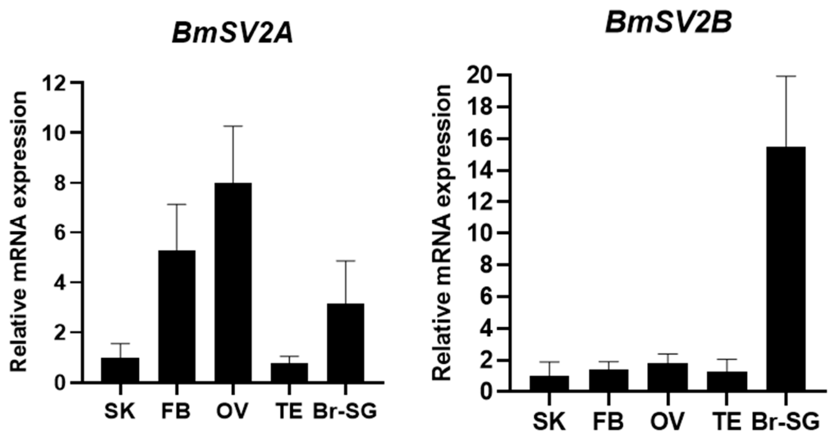

Figure S4. Tissue expression profiles of *BmSV2A* and *BmSV2B* genes. SK: skin; FB: fat body; OV: ovary; TE: testis; Br-SG: brain-suboesophageal ganglion.

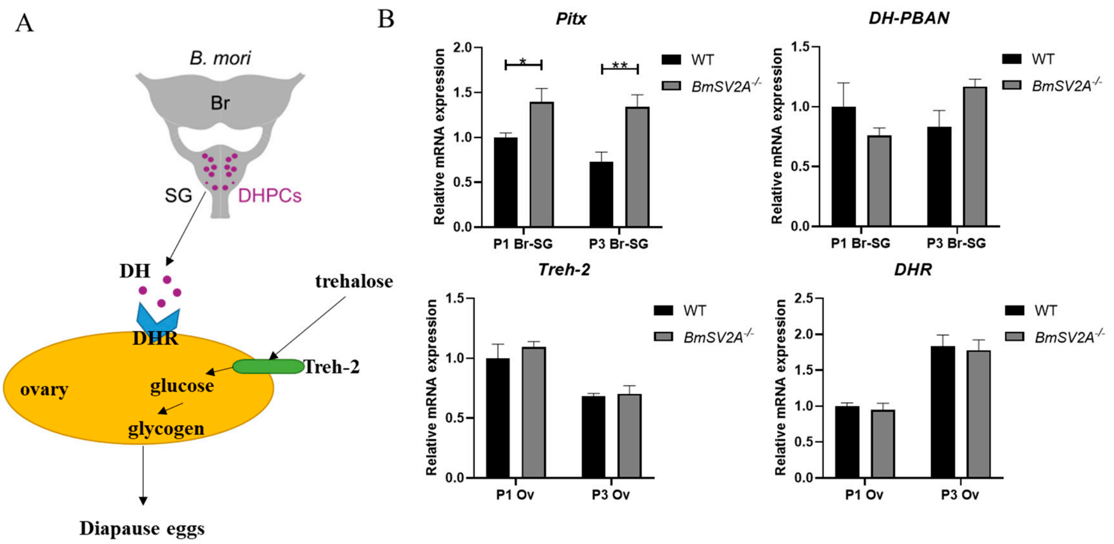

Figure S5. Expression of genes related to the synthesis and action pathways of diapause hormone. (**A**) Schematic diagram of diapause decision in the silkworm. (**B**) qRT-PCR analysis of *BmSV2A*. RNA were collected from 1-day-old and 3-day-old pupae. Br-SG: brain-suboesophageal ganglion. Ov: ovary. *, ** indicate that the *p*-values of *t*-tests are less than 0.05, 0.01, respectively. Error bars represent mean ± SE.

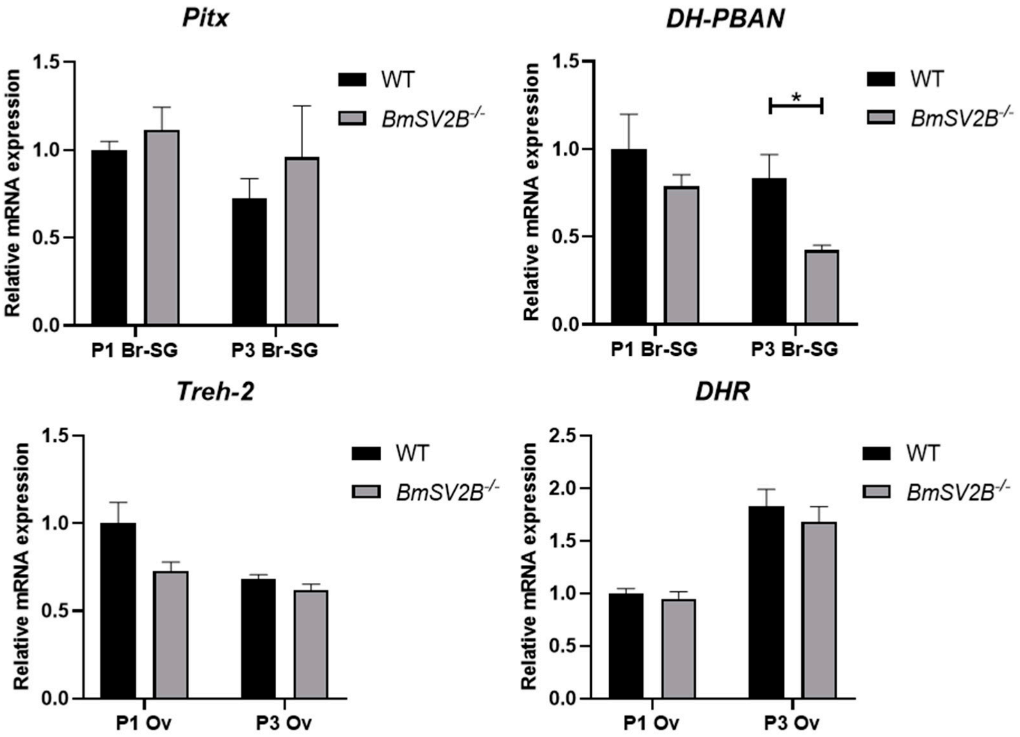

Figure S6. Expression of genes related to the synthesis and action pathways of diapause hormone. qRT-PCR analysis of *BmSV2B*. RNA were collected from 1-day-old and 3-day-old pupae. Br-SG: brain-suboesophageal ganglion. Ov: ovary. * indicate that the *p*-values of *t*-tests are less than 0.05. Error bars represent mean ± SE.

Tables S1–S4

Table S1. Primers for qRT-PCR

Gene name	Primer	Sequence (5' to 3')
DH-PBAN	DH-PBAN-F	AGCGATCAATGAAGCCATCCACTG
	DH-PBAN-R	TGCCTCTCGTAAGGTAGCTGGTC
DHR	DHR-F	GACACCGCAAATGCTTCG
	DHR-R	ACCCAATAACCCTGATACAAATA
Pitx	Pitx-F	GGTGTTGCTCCGTGCCCTTAC
	Pitx-R	TGATGAGTGCTGCTTCGCCTTC
Treh-2	Treh-2-F	GTGTCGTTGCTGATCGTAGCA
	Treh-2-R	GCCCGTGGCAGTAAATCATAC
GAD	GAD-F	TACCAAGTCAAAACCGGGCA
	GAD-R	ATCAAGATGAAGACCGGGGC
GRD	GRD-F	TTATCCTTGGCAGCTACGCC
	GRD-R	CGGTGTGCCTTTTGAGGTTG
RDL1	RDL1-F	TCCAGAATGCCCTCCAG
	RDL1-R	AAAACGGACTTCAGATGGTCT
RDL2	RDL2-F	TACCACCTAGCCGATCTTCG
	RDL2-R	TTGTCCTCCTGCTTCTTCGT
RDL3	RDL3-F	TTCGCTACAAGGTCCGAGAT
	RDL3-R	GTTCATCCTGCTCCTGCTG
LCCH3	LCCH3-F	ATCCACTCGACAGCCAGAAC
	LCCH3-R	TGGGGTAGTTCAGCGTCTTC
Ssadh	Ssadh-F	TGAATTTGGCATGGTTGCTA
	Ssadh-R	CCTTCACGCCCTATACCAGA
GABAT	GABAT-F	CAAGACCGGAAAGGTTTTGA
	GABAT-R	CAAGACCGGAAAGGTTTTGA
VGAT	VGAT-F	GGATGGAATTGCTCCAAGAA
	VGAT-R	TTTTAGCTCCTGGCTTTCCA
GAT	GAT-F	TCTTCAATCTCGTGCAGTGG
	GAT-R	GTGACACGCCACAGGTACAC
BmSV2A	BmSV2A-F	CGGCTTGTCCTCCACTAATC
	BmSV2A-R	CCGTCACAGTTCCCATATCC
BmSV2B	BmSV2B-F	GATACGTCAGTGCGTTGCAT
	BmSV2B-R	CCGAGTGTTGACGCTCTGTA
Rp49	Rp49-F	AGGCATCAATCGGATCGCTATG
	Rp49-R	TTGTGAACTAGGACCTTACGGAATC

Table S2. SgRNA target sequences and PCR detection primers for each knockout line

Knockout line	Target gene	Target sequence<br>20 mer+PAM (total 23 mer)	Primers for sgRNA target site detection
BmSV2A-360	*BmSV2A*	>*BmSV2A*-target1<br>CGTCGAGCAGGGCCCAGGCG<br>**AGG**<br>>*BmSV2A*-target2<br>GGTCTGGTGCCCCTTCTCAC<br>**GGG**	> BmSV2A _KO-F<br>TGAGCAGCCTGTTCTACACG<br>> BmSV2A _KO-R<br>TCGGTACGAATGCAACGATA
BmSV2B-263<br>BmSV2B-430	*BmSV2B*	>*BmSV2B*-target1<br>GGTGAGGCGGCCCTTGTCGG<br>**AGG**<br>>*BmSV2B*-target2<br>TTCGGAATCATCGGAGCCAC<br>**GGG**	> BmSV2B _KO-F<br>GATACGTCAGTGCGTTGCAT<br>> BmSV2B _KO-R<br>CACGTGTACTGCTCGCTGTT

Table S3. Analysis materials for population genetic differentiation index *F*st

Voltinism	Sample ID	Voltinism	Sample ID	Voltinism	Sample ID
1	Sample32A	2	Sample113A	poly-	Sample862A
1	Sample26A	2	SampleB49A	poly-	Sample885A
1	Sample458A	2	SampleB117B	poly-	Sample861A
1	Sample328A	2	Sample210A	poly-	Sample887A
1	Sample866A	2	SampleB7A	poly-	Sample859A
1	Sample8A	2	Sample223A	poly-	Sample879A
1	Sample171A	2	Sample218A	poly-	Sample892A
1	Sample623A	2	SampleB12A	poly-	Sample860A
1	Sample181A	2	Sample134A	poly-	Sample890A
1	Sample1A	2	SampleB6A	poly-	Sample888A
1	Sample595A	2	Sample269A	poly-	Sample165A
1	Sample52A	2	Sample597B	poly-	Sample148A
1	Sample34A	2	Sample125A	poly-	Sample886A
1	Sample44A	2	SampleB42A	poly-	Sample893A
1	Sample50A	2	Sample216A	poly-	Sample864-1A
1	Sample36A	2	SampleB11A	poly-	Sample889A
1	Sample28A	2	Sample217A	poly-	Sample864-2A
1	Sample42A	2	SampleB134A	poly-	Sample863A
1	Sample38A	2	SampleB133A		
1	Sample187A	2	SampleB9B		
1	Sample185A	2	SampleB50A		
1	Sample46A	2	SampleB47A		
1	Sample40A	2	Sample748A		
1	Sample15A	2	Sample215A		
1	Sample868A	2	SampleB139B		
1	Sample173A	2	Sample123A		
1	Sample161A	2	SampleB138B		
1	Sample285A	2	SampleB245A		
1	Sample330A	2	SampleB208A		
1	Sample341A	2	SampleB247A		
1	Sample16A	2	Sample145A		
1	Sample283A	2	Sample139A		
1	Sample234A	2	Sample206A		
1	Sample335A	2	SampleB309A		
1	Sample411A	2	Sample142A		
		2	SampleB323A		
		2	SampleB211A		
		2	SampleB241A		
		2	SampleB212A		
		2	SampleB209A		
		2	SampleB201A		

Voltinism	Sample ID	Voltinism	Sample ID	Voltinism	Sample ID
		2	Sample124A		
		2	SampleB311A		
		2	Sample138A		
		2	Sample136A		
		2	SampleB215A		
		2	SampleB343A		
		2	SampleB307B		
		2	Sample209A		
		2	Sample857A		
		2	SampleB308A		
		2	SampleB341A		
		2	Sample143A		
		2	Sample207A		
		2	SampleB337A		
		2	SampleB207A		

Table S4. Candidate region encoding protein genes and functional annotations

Gene *ID*	Name	Annotation
KWMTBOMO03142	H2B	late histone H2B.L4
KWMTBOMO03143	SV2B	synaptic vesicle glycoprotein 2B
KWMTBOMO03144	SV2A	synaptic vesicle glycoprotein 2A
KWMTBOMO03145	SMAD6	mothers against decapentaplegic homolog 6
